# Genome-scale molecular profiling from routine histopathology with a multimodal pathology foundation model

**DOI:** 10.21203/rs.3.rs-9949149/v1

**Published:** 2026-08-06

**Authors:** Qing Li, Jian Sang, Yiwei Xiao, Pengzhi Zhang, Weiqing Chen, Tu N. Tran, Zejuan Li, Shengyu Li, Tongwu Zhang, Guangyu Wang

**Affiliations:** 1. Center for Bioinformatics and Computational Biology, Houston Methodist Research Institute, Houston, TX, 77030, USA; 2. Center for Cardiovascular Regeneration, Houston Methodist Research Institute, Houston, TX, 77030, USA; 3. Center for RNA Therapeutics, Houston Methodist Research Institute, Houston, TX, 77030, USA; 4. Department of Cardiothoracic Surgery, Weill Cornell Medicine, Cornell University, New York, NY, 10065, USA; 5. Department of Physiology, Biophysics & Systems Biology, Weill Cornell Graduate School of Medical Science, Cornell University, New York, NY, 10065, USA; 6. Department of Pathology and Genomic Medicine, Houston Methodist Hospital, Houston Methodist Research Institute, Houston, TX, USA; 7. Division of Cancer Epidemiology and Genetics, National Cancer Institute, Bethesda, MD, USA

## Abstract

Pathology foundation models trained from whole-slide images alone treat tissue morphology as an autonomous visual phenotype, leaving learned representations only weakly anchored to the molecular processes that generate tissue architecture. Here we present Fuji, a multimodal pathology foundation model that grounds morphology in transcriptomic and mutational supervision through a two-stage pretraining strategy: multi-teacher contrastive distillation that harmonizes formalin-fixed paraffin-embedded and fresh-frozen specimens, followed by multimodal multi-task learning that jointly optimizes masked image reconstruction, transcriptomic embedding reconstruction, and attention-aligned mutational signature inference. Trained on 60,546 whole-slide images paired with 45,675 bulk RNA-seq profiles and 22,683 whole-genome sequencing samples drawn from TCGA, GTEx, and CPTAC, Fuji yields a protocol-invariant, genome-informed embedding that we treat as a quantitative coordinate system for probing how genomic states project into tissue architecture. Genome-scale instability phenotypes, microsatellite instability, chromosomal instability, homologous recombination deficiency, and whole-genome duplication, are recoverable from histology with AUCs up to 0.98, consistently exceeding existing pathology foundation models. Extrachromosomal DNA amplification leaves a distinct morphological footprint that stratifies survival independently of tissue lineage. Etiologic mutational processes such as tobacco exposure and mismatch repair deficiency localize to discrete architectural neighborhoods, validated in two independent population-based cohorts, whereas clock-like signatures remain diffuse, delineating the scope and limits of genomic visibility from routine histology. Fuji further enables direct inference of tumor mutational burden, neoantigen load, immunotherapy response, and FDA-actionable driver mutations from hematoxylin and eosin slides. By coupling pretraining to molecular and mutational supervision, Fuji establishes an engineering basis for genome-informed inference from the most widely performed assay in oncology.

## Introduction

Tumor genome evolution shapes tissue architecture, but the extent to which this coupling is quantitatively recoverable from routine histopathology remains unclear. Tissue morphology is not merely a visual phenotype: it reflects the integrated consequences of cell-intrinsic regulatory programs, somatic genomic alterations, mutational processes, and microenvironmental interactions accumulated over the evolutionary history of the tumor^1–10^. Clonal selection, genomic amplification, and mutagenesis alter growth dynamics, stromal organization, and spatial constraints in ways that ultimately manifest as the structural patterns observed in hematoxylin and eosin (H&E) slides^11–21^. Pathologists exploit a fraction of this signal at the level of subtype and grade, but whether broader classes of genomic information, instability phenotypes, focal structural amplifications, mutational exposures, and driver alterations, leave systematic and quantitatively recoverable architectural correlates has not been established within a unified framework. The practical stakes are substantial: comprehensive genomic profiling now guides therapy selection, prognostic stratification, and clinical trial eligibility^22–24^, yet cost, tissue requirements, and infrastructure restrict sequencing to a fraction of patients worldwide, while H&E slides are produced on virtually every resected tumor. A method that systematically maps genome state to histology would therefore both advance the biology of tumor architecture and provide a scalable engineering route to genomically informed cancer analysis.

Recent pathology foundation models have substantially advanced whole-slide image representation, demonstrating strong performance in diagnostic classification, prognosis prediction, and selected biomarker inference^4–10^. Their pretraining objectives, however, are vision-only, image-image self-distillation, masked autoencoding, or contrastive learning on tiles, and vision-language extensions align morphology with coarse clinical or textual descriptors rather than with the molecular processes that generate tissue architecture^16,17,25–27^. Embeddings learned under these objectives are powerful representations of visual phenotype but only weakly anchored to the functional and evolutionary determinants of morphology, limiting their capacity to recover latent genomic states that are only indirectly visible at histologic resolution. A second technical disconnect compounds this gap: pretraining and benchmarking have largely been performed on formalin-fixed paraffin-embedded (FFPE) or fresh-frozen specimens separately, even though clinical pathology is dominated by FFPE tissue while molecular profiling has historically relied on fresh-frozen specimens. The result is a systematic mismatch between the modality that carries molecular supervision and the modality in which routine pathology is practiced. Whether genome-scale instability, focal amplification mechanisms, etiologic mutational processes, and clinically actionable driver mutations are jointly recoverable from a unified, protocol-invariant pathology representation has not been established.

Here we present Fuji, a multimodal pathology foundation model that addresses this gap by grounding tissue representation learning in transcriptomic and mutational supervision during pretraining. The system is built around two engineering decisions. First, rather than retraining a backbone from scratch, we combine two state-of-the-art pathology foundation models, GigaPath which provides whole-slide morphological context^4^, and CHIEF which contributes regional and semantic structure^5^, through multi-teacher contrastive distillation, freezing most of the backbone and training only the top representation layer. Paired FFPE and fresh-frozen sections from the same patient are explicitly aligned within this latent space to suppress protocol-specific variation. Second, the distilled backbone is refined through a multimodal multi-task objective with three components: masked reconstruction of patch-level image features preserves fine-grained morphology; reconstruction of paired transcriptomic embeddings couples visual representation to molecular state; and a multi-head attention module classifies mutational signatures under an attention-alignment constraint that ties signature-specific attention to histologically informative regions, providing spatial grounding for genomic supervision. Mutational supervision is staged from whole-exome-derived signatures to higher-resolution whole-genome-derived signatures.

We trained Fuji on 60,546 whole-slide images from 29 tissue types drawn from TCGA, GTEx, and CPTAC^28–30^, paired with 45,675 bulk RNA-seq profiles and 22,683 whole-genome sequencing samples, and built a modular ecosystem of task-specific decoders on top of the resulting frozen embedding. Treating this embedding as a quantitative coordinate system, we find that genomic states project into it non-randomly. Microsatellite instability, chromosomal instability, homologous recombination deficiency, and whole-genome duplication are recoverable with AUCs up to 0.98 across FFPE and fresh-frozen cohorts, consistently exceeding existing pathology foundation models. Extrachromosomal DNA amplification produces a distinct architectural footprint that stratifies overall survival in lower-grade glioma and endometrial carcinoma independently of tissue lineage. Tobacco exposure and mismatch repair deficiency localize to discrete morphological neighborhoods reproduced in two independent population-based cohorts (HCMI and PLCO), while clock-like signatures remain diffuse. Fuji additionally infers tumor mutational burden, neoantigen load, immunotherapy response, and FDA-actionable driver mutations directly from H&E slides.

## Results

### Fuji: a genome-informed multimodal pathology foundation model

**Fig. 1.**
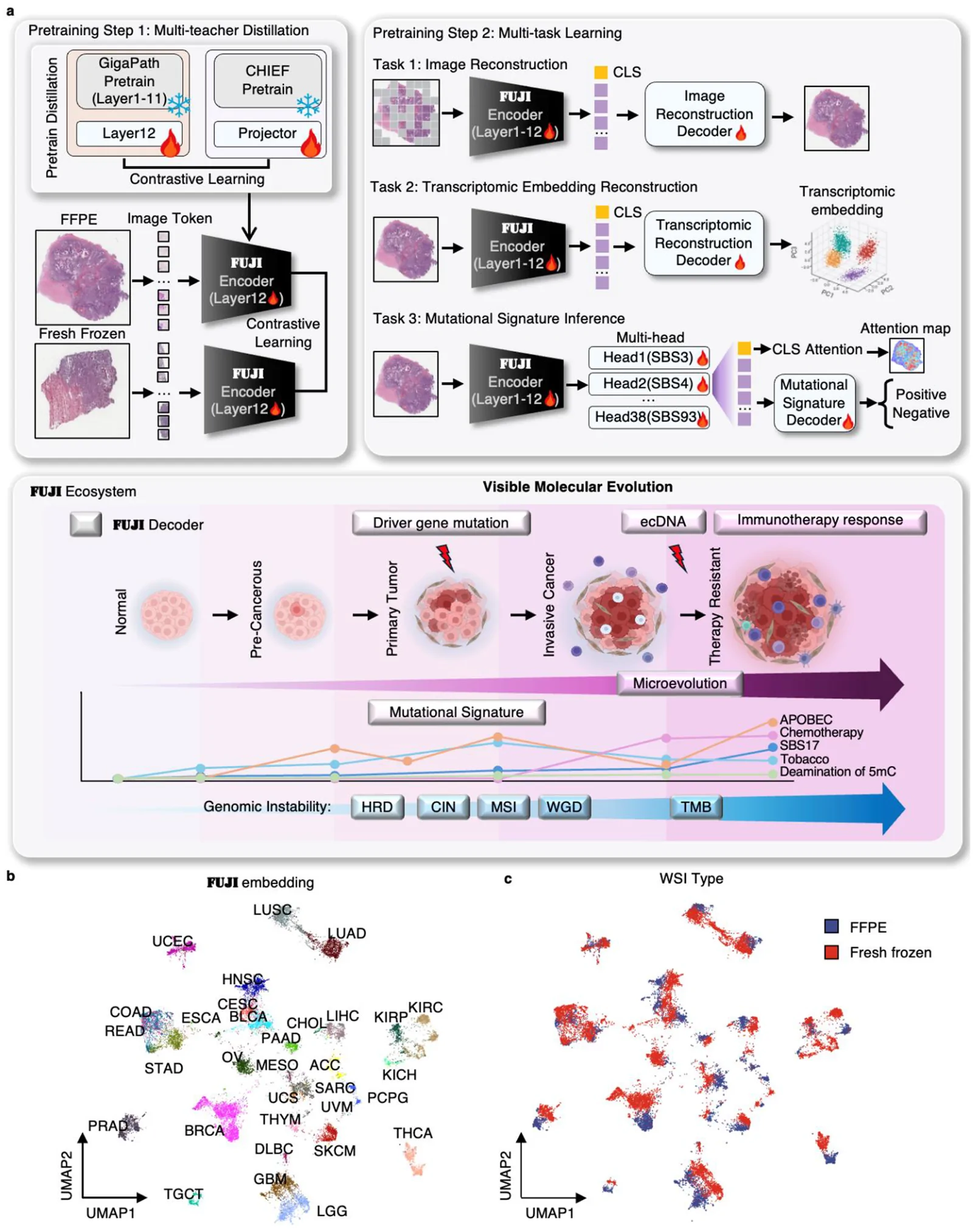
Overview of the Fuji foundation model architecture, multi-modal training strategy, and decoder ecosystem. **a**, Two-step pretraining framework for the Fuji encoder. In Step 1, the model utilizes multi-teacher distillation to align features from FFPE and fresh-frozen slides with contrastive learning. A dedicated Layer 12 and projector are optimized to integrate diverse morphological signals in pre-trained representations from GigaPath (layers 1–11) and CHIEF. In Step 2, the encoder is jointly optimized three tasks: task 1 is masked image reconstruction to capture fine-grained morphological details; task 2 performs transcriptomic embedding reconstruction to map visual phenotypes to molecular expression profiles; and task 3 executes mutational signature inference via a multi-head attention mechanism (e.g., SBS3, SBS4, SBS93), producing signature-specific attention maps and binary classification outputs. The Fuji decoder ecosystem translates genome-informed morphology embeddings into clinically relevant genomic readouts across the full arc of tumor evolution. Upper panel: key genomic events are anchored to progressive disease stages, driver gene mutations at primary tumor formation, extrachromosomal DNA amplification during invasion, and immunotherapy response at therapy resistance. Lower panel: genomic instability phenotypes (HRD, CIN, MSI, WGD, and TMB) accumulate across the evolutionary continuum, while mutational signatures (APOBEC, chemotherapy, SBS17, tobacco, and deamination of 5mC) reflect stage-specific etiologic history. **b**, UMAP projection of slide-level embeddings across the pan-cancer. Each point represents a single WSI, colored by cancer type. The clustering demonstrates the model’s ability to preserve biological variance and capture lineage-specific morphological signatures without explicit supervision. **c**, Harmonization of embeddings across tissue preparations. Visualization of embedding harmonization between FFPE (blue) and fresh-frozen (red) tissue preparations.

We developed Fuji, a multimodal pathology foundation model that learns representations of human tissue by jointly integrating whole-slide images with transcriptomic profiles and mutational signatures derived from genomic sequencing (Fig. 1a). Fuji adopts a two-stage pretraining strategy that couples robust morphological representation learning with explicit molecular and genomic grounding. In the first stage, we established a domain-general histological backbone using image-only multi-teacher distillation. Two state-of-the-art pathology foundation models, GigaPath and CHIEF, were aligned within a unified contrastive framework: GigaPath contributes high-capacity whole-slide morphological representations, while CHIEF provides complementary semantic and regional contextual features. We froze the GigaPath backbone (layers 1–11) and optimized only the top representation layer (layer 12) of the Fuji encoder, introducing a lightweight projection head on top of the frozen CHIEF encoder to map its embeddings into the shared latent space. Paired FFPE and fresh-frozen sections from the same patients were additionally incorporated to explicitly model systematic differences in color, texture, and artifact profiles arising from tissue processing.

In the second stage, Fuji was refined through a multimodal multi-task learning framework that grounds morphology in molecular and genomic signals. Three objectives were jointly optimized: reconstruction of masked pathology image features to preserve fine-grained morphological information, reconstruction of transcriptomic embeddings to align visual representations with mutagenic processes. Mutational signatures were inferred from whole-exome sequencing (WES) during an initial training phase and subsequently refined using whole-genome sequencing (WGS), enabling progressive supervision with increasing genomic resolution. To ensure spatial grounding of genomic supervision, we further encouraged alignment between Fuji’s attention maps and those learned by a multiple-instance learning model trained for mutational signature prediction, guiding Fuji to localize genomic signals to histologically relevant tissue regions.

Fuji was trained on 60,546 WSIs from 29 major tissue types, including 42,351 FFPE and 18,195 fresh-frozen slides, drawn from the Cancer Genome Atlas (TCGA, 29,834 WSIs)^28^, the Genotype-Tissue Expression project (GTEx, 24,522 WSIs)^29^, and the Clinical Proteomic Tumor Analysis Consortium (CPTAC, 6,190 WSIs)^30^. These images were paired with 45,675 bulk RNA-seq profiles, 31,544 WES samples, and 22,683 WGS samples, providing increasingly comprehensive mutational information across pretraining phases (Extended Data Fig. 1a).

Building on the pretrained encoder, we developed a modular ecosystem of task-specific decoders that operate on a shared, genome-informed whole-slide representation (Fig. 1a, Extended Data Fig. 1b). This ecosystem translates Fuji embeddings into diverse genomic and clinical readouts, including mutational features, genomic instability phenotypes, therapeutic response, and tissue-level annotations, without requiring retraining of the foundation model. To assess the biological structure of the learned representations, we examined the organization of Fuji’s embedding space through low-dimensional projection. WSIs grouped clearly by cancer type, with closely related malignancies forming coherent neighborhoods (Fig. 1b). Critically, FFPE and fresh-frozen slides from the same tissue types were well aligned and intermingled within the same clusters (Fig. 1c, Extended Data Fig. 2), indicating that Fuji effectively suppresses protocol-specific variation while preserving disease- and tissue-relevant morphology. In contrast, embeddings derived from GigaPath and CHIEF exhibited partial mixing of distinct cancer types, with FFPE and fresh-frozen slides forming largely separated clusters (Extended Data Fig. 3), reflecting strong sensitivity to tissue processing artifacts rather than intrinsic histological similarity. Together, these analyses establish Fuji’s embedding as a biologically structured, protocol-invariant coordinate system of tissue morphology, enabling consistent representation of tissue identity across specimen preparation modalities, a long-standing barrier to translating molecularly grounded pathology models into clinical practice.

### Morphology embeddings capture neoplastic progression and clonal evolution

**Fig. 2.**
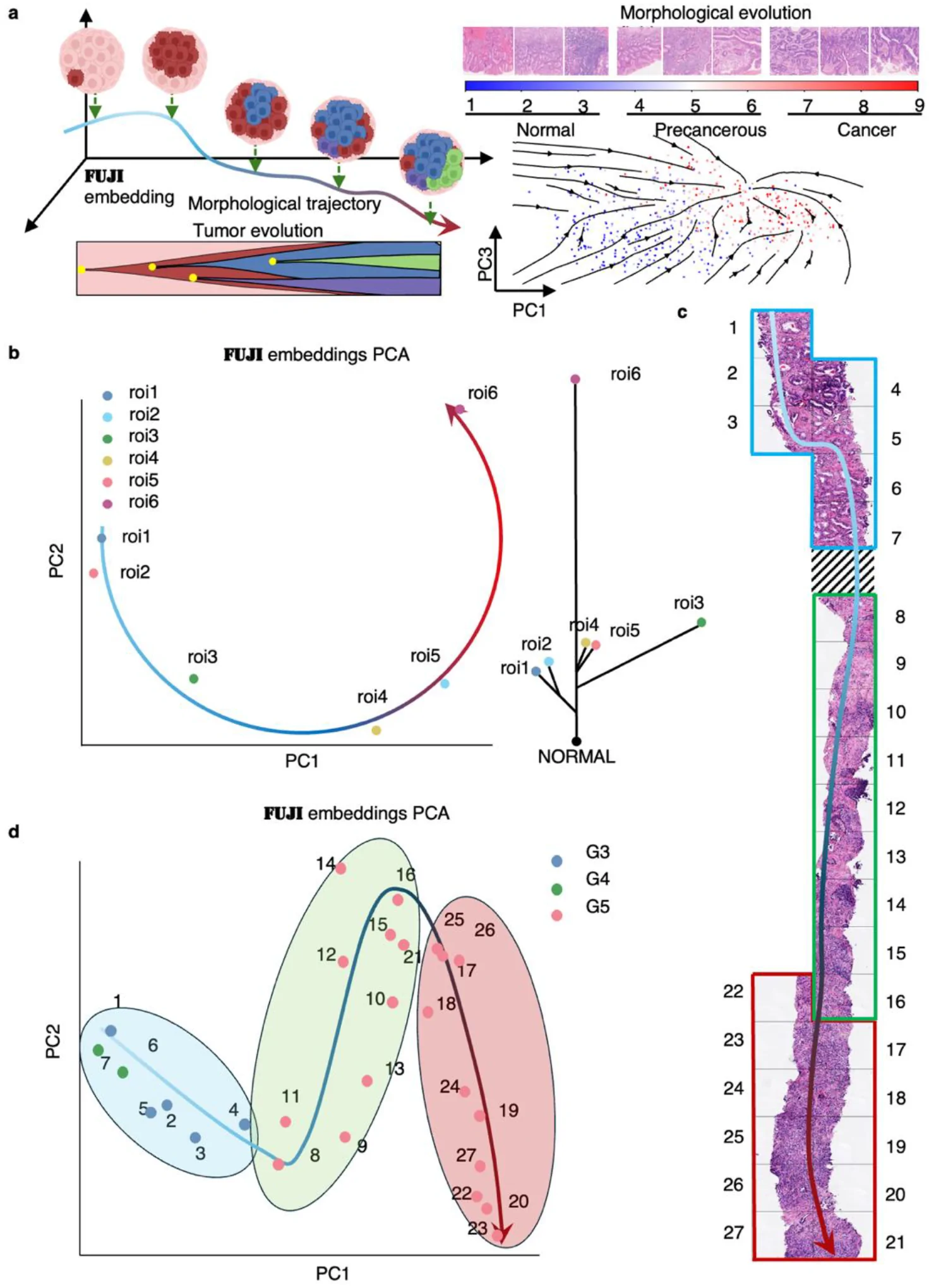
The Fuji foundation model pan-cancer morphological evolution field. **a**, The morphological evolution field and directed progression manifold. (Left-Top) Standardized progression sequence from normal mucosa through precancerous lesions to invasive carcinoma. (Left-Bottom) Vector field analysis on the PCA projection reveals a continuous, directed manifold that captures the global trajectory of malignant progression. Right morphological evolution field H&E patches (Right-Top) illustrating the transition from normal (stages 1–3) to precancerous (4–6) and cancer (7–9) states. (Right-Bottom) PCA projection of Fuji embeddings for 600 annotated regions. The directed vector field captures the continuous progression trajectory inferred via graph-based pseudotime. **b**, Vectors indicate the direction and magnitude of colorectal morphological divergence from normal tissue, highlighting progressive deviation toward malignant states. **c**, Prostate histology sampled along this axis shows stepwise architectural disruption, supporting embedding-based analysis of intra-patient tumor evolution. **d**, Regions from a single prostate biopsy form an ordered path in morphology embedding space, defining a within-patient progression axis.

To test whether Fuji’s embedding behaves as a continuous coordinate system rather than a discrete classifier output, we examined whether unsupervised trajectory inference on the embedding recovers biologically meaningful progression structure. We performed diffusion-based trajectory modeling on 600 expert-annotated gastric tissue regions spanning nine pathological stages: 257 normal mucosa, 174 precancerous lesions (including chronic atrophic gastritis and intestinal metaplasia), and 169 invasive carcinomas (Fig. 2a). Diffusion pseudotime analysis applied to slide-level embeddings inferred a continuous progression axis without using histologic stage labels. The resulting trajectory exhibited structured ordering, with normal samples localized toward one extreme, precancerous lesions occupying intermediate positions, and invasive carcinomas enriched at the opposite end (Fig. 2a). Representative histologic tiles sampled along this axis demonstrated progressive architectural disruption, including increasing glandular irregularity and stromal expansion. Vector field analysis on the embedding projection revealed a continuous directed manifold capturing the global trajectory of malignant progression, indicating that morphological transformation is not merely categorizable but quantitatively orderable within the embedding space.

We next asked whether the embedding can resolve clonal evolution within individual tumors. In a colorectal cancer patient with multi-region sampling, regions of interest traced a continuous path in morphological embedding space (Fig. 2b). The ordering was supported by an independent WES-derived evolutionary phylogeny constructed from the same regions (Fig. 2b), indicating that patient-specific genetic progression aligns with a coherent trajectory of tissue architectural change. Similar within-patient structure was observed in a prostate biopsy, where spatially distinct regions formed an ordered trajectory from near-normal glands to invasive tumor morphology (Fig. 2c, d). These results establish that Fuji embeddings function as a quantitative coordinate system in which both cross-cancer neoplastic progression and within-patient clonal evolution are geometrically organized, providing a foundation for interpreting downstream genomic inference as reflecting biologically coherent architectural states rather than isolated visual correlations.

### Genome-scale instability phenotypes as clinically actionable readouts from routine histology

**Fig. 3.**
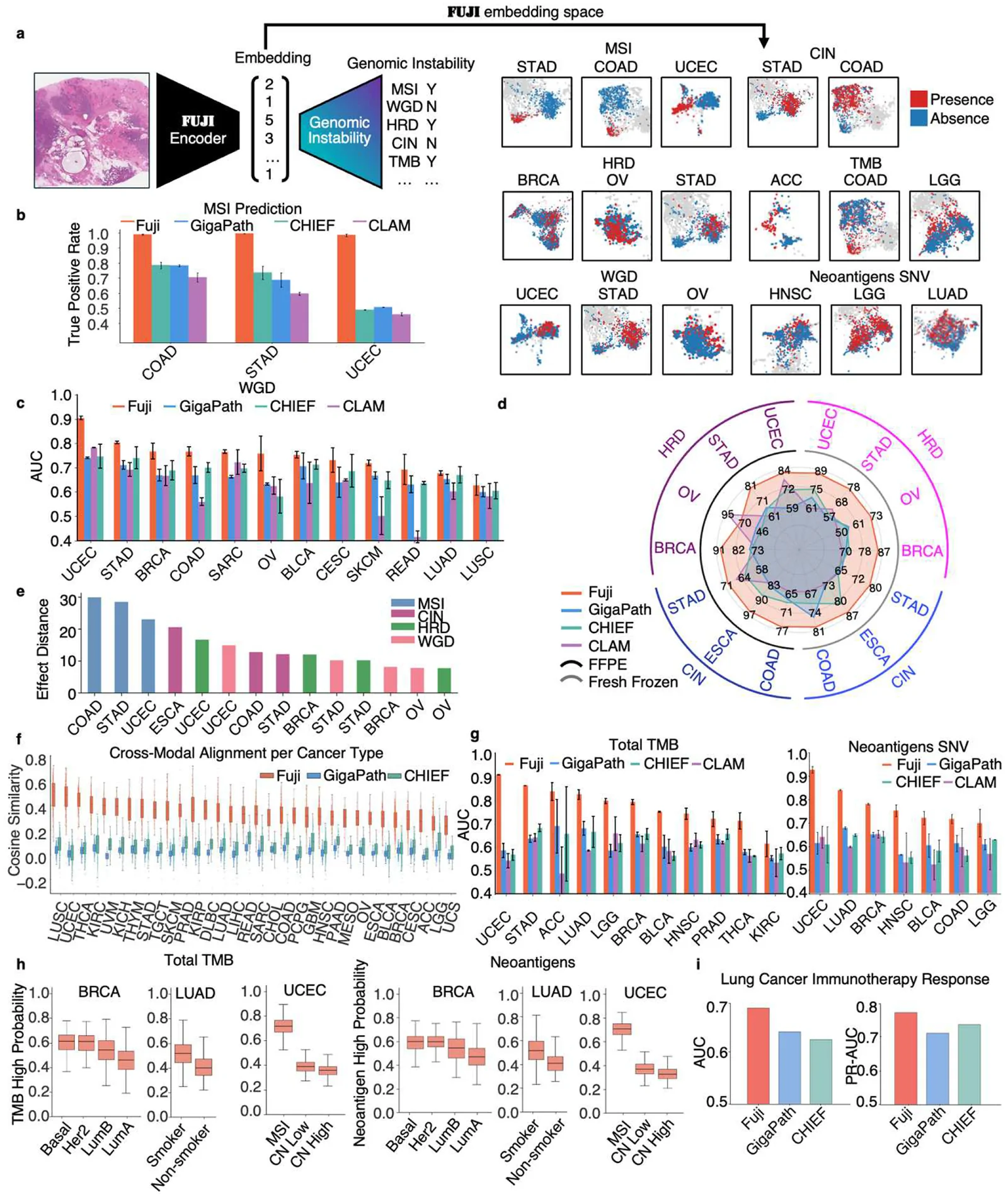
Prediction of genomic instability, tumor mutational burden, neoantigen load, and immunotherapy response from whole-slide images. **a**, (Left) Inference of global genome-scale hallmarks. Schematic of the Fuji encoder-based framework for predicting quantitative genomic instability and mutational loads directly from WSIs. Fuji maps each slide to a shared, genome-informed embedding, allowing for the interrogation of diverse molecular phenotypes, including MSI, CIN, HRD, TMB, WGD, neoantigens SNV through task-specific decoders. (Right) Lineage-anchored localization of instability phenotypes. Binary localization of genomic instability hallmarks in the embedding manifold, showing distinct morphological regions associated with molecular presence (red) versus absence (blue). **b**, Bar plots comparing the AUC of Fuji and baseline models for the prediction of MSI across various cancer types. AUC values with 95% confidence intervals are indicated. Error bars represent the standard deviation between FFPE and fresh-frozen specimens. **c**, Bar plots comparing the AUC of Fuji and baseline models for WGD prediction from WSIs across multiple cancer types. Results are shown for both FFPE and fresh-frozen specimens. Fuji is compared with GigaPath, CHIEF, and CLAM. Error bars indicate the standard deviation across FFPE and fresh-frozen evaluations. **d**, Radar plots summarizing model performance for the prediction of CIN and HRD from H&E-stained WSIs across cancer types. Results are shown for FFPE (inner ring) and fresh-frozen (outer ring) specimens. Fuji is compared with GigaPath, CHIEF, and CLAM. Numbers indicate AUC values (%). **e**, Magnitude of genomic instability associated with morphology imprint. Effect distance analysis measures the geometric magnitude of embedding shifts between high instability and low instability tumors within each lineage. **f**, Boxplots illustrating the cosine similarity between paired fresh-frozen and FFPE whole-slide image embeddings across 32 TCGA cancer types, comparing the Fuji encoder (red), GigaPath (blue), and CHIEF (green). **g**, Bar plots comparing the AUC of Fuji and baseline models for the prediction of total tumor mutational burden (TMB) and neoantigen SNV across various cancer types. Error bars represent the standard deviation between FFPE and fresh-frozen specimens. **h**, Box plots showing Fuji-predicted probabilities for total TMB and neoantigen SNV, stratified by established clinical and molecular subtypes (BRCA subtypes, LUAD smoking status, UCEC molecular subtypes). **i**, Bar plots comparing model performance for immunotherapy response prediction in lung cancer. Left and right panels show AUC and PR-AUC, respectively.

Genome-scale instability phenotypes, microsatellite instability (MSI), chromosomal instability (CIN), homologous recombination deficiency (HRD), and whole-genome duplication (WGD), are integrative readouts of tumor evolutionary history that reshape nuclear morphology, stromal organization, and immune infiltration through well-characterized mechanistic routes. They therefore constitute a stringent test of whether a molecularly grounded embedding captures features that vision-only pretraining cannot. We trained lightweight decoders on frozen Fuji embeddings to infer each instability phenotype from routine H&E slides, using sequencing-derived labels as supervision, and compared against decoders trained on embeddings from CHIEF, GigaPath, and CLAM (Fig. 3a).

We first assessed MSI prediction in stomach adenocarcinomas (STAD), colorectal (COAD), and uterine corpus endometrial (UCEC), three tumor types in which MSI represents a major molecular subtype with established therapeutic implications for immune checkpoint blockade. Across all three cancer types and both FFPE and fresh-frozen specimens, Fuji consistently achieved area under the receiver operating characteristic curve (AUC) values of 0.96–0.98 (Fig. 3b), substantially outperforming CHIEF, GigaPath, and CLAM baselines, which ranged from 0.65 to 0.83. In COAD, Fuji achieved an AUC of 0.97 in both FFPE and fresh-frozen cohorts, exceeding baseline performance by 0.14–0.25 AUC. In UCEC, absolute improvements exceeded 0.39 AUC over competing methods. The strong performance of Fuji in MSI prediction was consistent with known biological correlates of mismatch repair deficiency, including enrichment of MSI-associated mutational signatures such as SBS44 and DBS7, as well as recurrent alterations in the mismatch repair genes MLH1, MSH2, MSH6, and PMS2 in both FFPE and fresh frozen tissue images (Extended Data Fig. 4).

We next evaluated WGD across 12 cancer types with high WGD prevalence^31^ (Fig. 3c). Fuji consistently achieved the highest AUC in every cohort compared with baseline models, with the largest gains observed in UCEC (mean AUC 0.91), STAD (mean AUC 0.81), and breast cancer (BRCA; mean AUC 0.76). For HRD prediction in UCEC, STAD, OV, and BRCA, tumor types in which homologous recombination defects drive genomic instability and have established therapeutic relevance for PARP inhibition^32,33^, Fuji achieved the highest predictive accuracy in all settings except ovarian FFPE, with AUC values ranging from 0.71 to 0.89 compared with 0.44 to 0.77 for baseline approaches (Fig. 3d). For CIN prediction, analyses focused on COAD, STAD, and esophageal carcinoma (ESCA), tumor types characterized by extensive somatic copy-number alterations^34–36^. Across all three cancer types and both FFPE and fresh-frozen specimens, Fuji consistently achieved the highest predictive accuracy, with AUC values of 0.70–0.95 (Fig. 3d).

To assess whether these instability phenotypes correspond to structured architectural states rather than merely to visually separable categories, we quantified embedding separation between instability-positive and instability-negative tumors within each lineage using effect distance analysis (Fig. 3e). MSI and CIN produced the largest and most consistent embedding shifts in gastrointestinal and endometrial cancers, while HRD and WGD showed measurable separation across multiple lineages, supporting a structured, reproducible coupling between instability state and tissue architecture. The high concordance of predictive performance between FFPE and fresh-frozen cohorts (Fig. 3f) further indicates that Fuji learns shared, protocol-invariant morphological features associated with instability status, a critical property for clinical deployment where FFPE dominates routine workflows.

### Tumor mutational burden, neoantigen load, and immunotherapy response prediction

Beyond genome-scale instability, tumor mutational burden and predicted neoantigen load are clinically established biomarkers of tumor immunogenicity and response to immune checkpoint blockade. We evaluated whether Fuji could infer these mutation-derived immunogenic features directly from WSIs across 11 cancer types (Fig. 3g). For TMB prediction, Fuji achieved the highest performance among evaluated models, with AUC values ranging from 0.58 to 0.91 and exceeding 0.70 in the majority of cancer types. Absolute AUC improvements over baselines typically ranged from 0.06 to 0.34, with the largest gains observed in UCEC, STAD, and adrenocortical carcinoma (ACC). For neoantigen single-nucleotide variant (SNV) prediction (Fig. 3g), Fuji similarly demonstrated robust performance across cancer types and tissue preparations, achieving AUCs of 0.66–0.94. Performance was particularly strong in immunologically relevant tumors, including UCEC (AUC 0.92–0.94), LUAD (AUC 0.83–0.84), and BRCA (AUC 0.78–0.79), whereas baseline models achieved AUCs of 0.44–0.68. Consistent results were observed for silent and non-silent TMB prediction (Extended Data Fig. 5).

To assess whether Fuji-derived predictions reflect biologically coherent axes of variation rather than generic grade- or tumor-purity-like effects, we examined predicted scores across established molecular subtypes (Fig. 3h). In BRCA, basal-like and HER2-enriched subtypes showed higher scores than luminal A and luminal B tumors^37^, consistent with their known elevated mutational loads. In LUAD, smokers exhibited higher predicted probabilities than non-smokers, aligning with established exposure-mutation relationships^38^. In UCEC, MSI tumors showed the highest scores compared with copy number–low and copy number–high subtypes, consistent with the hypermutator phenotype of mismatch repair–deficient endometrial cancers^39^. These subgroup-specific patterns indicate that Fuji captures the molecular substructure of these phenotypes rather than a nonspecific tumor signal.

Given the established association between TMB, neoantigen load, and response to immune checkpoint blockade^40^, we next evaluated whether Fuji embeddings could predict clinical response to immunotherapy in a cohort of lung cancer patients who received immune checkpoint inhibitor therapy (Fig. 3i). Fuji achieved the highest performance among evaluated models, with an AUC of 0.70 and a PR-AUC of 0.79, outperforming GigaPath (AUC 0.64, PR-AUC 0.71) and CHIEF (AUC 0.63, PR-AUC 0.74). These findings suggest that Fuji captures histomorphological features linked to tumor immunogenicity and treatment response that are not fully represented in existing pathology foundation models. Given the modest cohort size, these results should be considered proof-of-concept, motivating prospective validation in larger and more diverse immunotherapy cohorts.

### Extrachromosomal DNA as a morphology-accessible prognostic biomarker

**Fig. 4:**
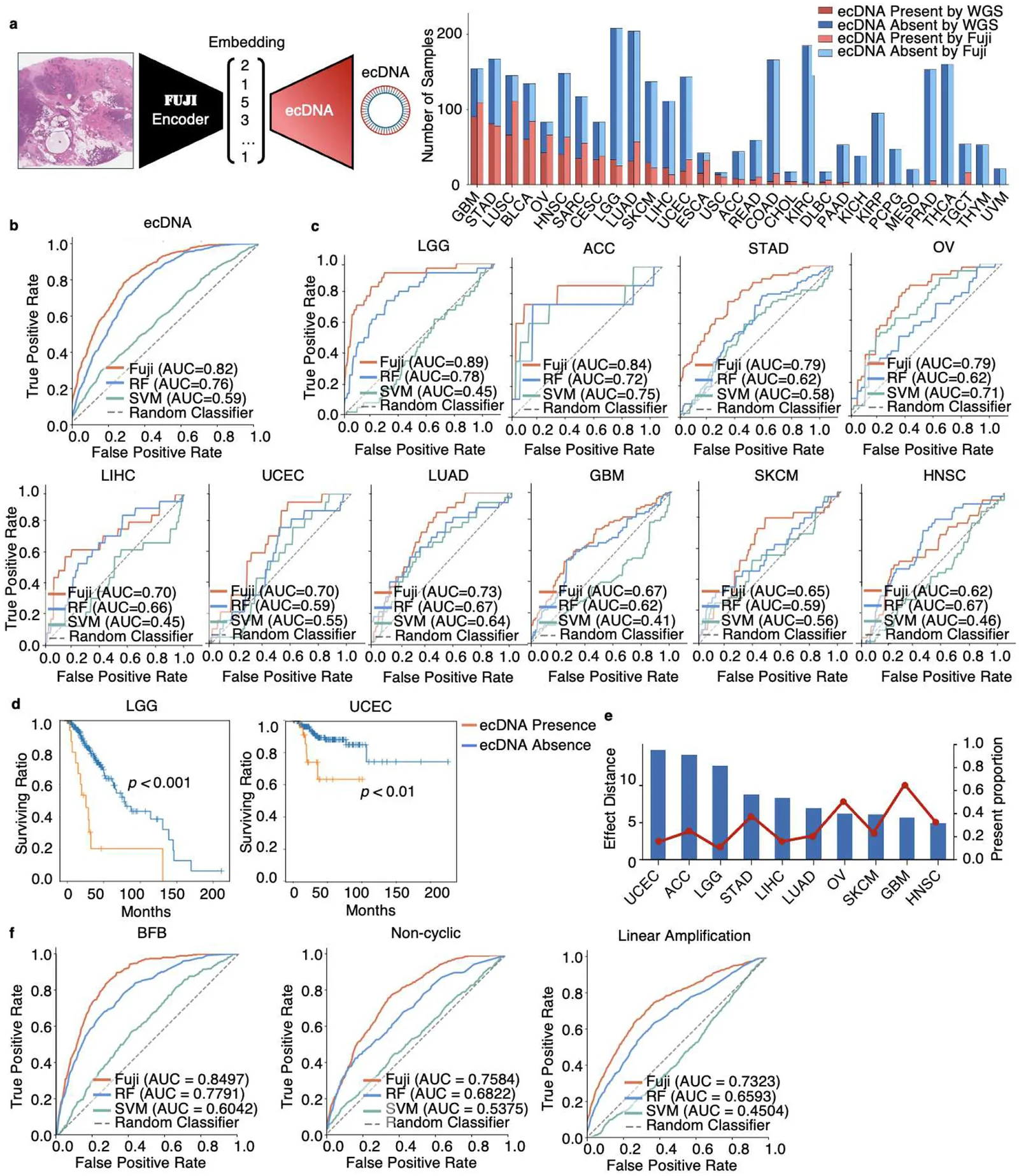
Detection of extrachromosomal DNA. **a,** (Left) Deciphering the architectural footprint of ecDNA. Implementation of a lightweight decoder on Fuji embeddings to infer ecDNA status, anchored in WGS-derived ground truth. (Right) Pan-cancer prevalence and identification of ecDNA. Stacked bar chart showing the distribution of ecDNA across 31 malignancy types. For each cancer type, the WGS-derived ground truth (dark red/blue) is compared with the Fuji decoder’s predictions (light red/blue). **b**, Pan-cancer performance of ecDNA detection. ROC curves comparing the Fuji decoder (blue) against non-neural baselines, including random forest (RF, green) and support vector machine (SVM, orange). **c**, Lineage-specific ROC analysis. Evaluation of model performance across ten representative cancer types. **d**, Prognostic impact of inferred ecDNA presence. Kaplan-Meier survival curves for patients with lower-grade glioma (LGG, left) and endometrial carcinoma (UCEC, right), stratified by the predicted presence (orange) or absence (blue) of ecDNA. **e**, Magnitude of ecDNA associated morphology imprint. Effect distance analysis quantifies the geometric magnitude of embedding separation between ecDNA present and ecDNA absent tumors within each lineage. The superimposed red trend line highlights the present proportion of ecDNA across cancer types. **f**, Gene amplification mechanisms prediction. AUC of BFB cycle detection, complex non-cyclic chromosomal rearrangement detection, and linear amplification pattern detection.

To investigate whether high-amplitude focal structural alterations project into structured geometric patterns within a genome-informed representation space, we examined the visibility of extrachromosomal DNA (ecDNA) within the Fuji latent manifold (Fig. 4a). Extrachromosomal DNA is a circular DNA–based amplification mechanism that drives high oncogene copy number, promotes intratumoral heterogeneity, and accelerates clonal evolution, and it has emerged as a marker of aggressive tumor biology with poor clinical outcomes^41^. Detection of ecDNA currently requires whole-genome sequencing, placing it beyond routine clinical assessment for most patients. We asked whether ecDNA status can be inferred directly from tissue morphology encoded in Fuji embeddings.

We developed a lightweight decoder trained to predict ecDNA status from slide-level embeddings, using WGS-derived ecDNA annotations as supervision. Across 31 cancer types in the TCGA held-out cohort, the Fuji ecDNA decoder achieved a pan-cancer AUC of 0.89 ± 0.06 (Fig. 4b), indicating robust discrimination between ecDNA-positive and ecDNA-negative tumors from histopathology alone. Statistical comparison of correlated ROC curves demonstrated that the Fuji decoder significantly outperformed support vector machine (AUC 0.82 ± 0.08) and random forest (AUC 0.84 ± 0.07) baselines trained on the same embeddings. Per-cancer-type analysis (Fig. 4c) revealed robust discrimination in tumors with high ecDNA prevalence, including lower-grade glioma (LGG; AUC 0.89), adrenocortical carcinoma (AUC 0.84), stomach adenocarcinoma (AUC 0.79), ovarian cancer (AUC 0.79), and LUAD (AUC 0.73), spanning diverse tissue origins and architectural contexts.

To evaluate the clinical relevance of morphology-derived ecDNA predictions, we performed survival analysis stratifying patients by inferred ecDNA status (Fig. 4d). In LGG and UCEC, tumors predicted to harbor ecDNA were associated with significantly worse overall survival compared with ecDNA-negative tumors (LGG, p<0.001; UCEC, p<0.01), consistent with the established association between ecDNA-driven oncogene amplification, aggressive tumor biology, and poor clinical outcomes^42^. These survival differences emerged despite ecDNA being inferred solely from histopathology, indicating that Fuji captures clinically meaningful morphological correlates of focal genomic amplification. To confirm that this signal reflects structural specificity rather than a generic malignancy axis, we quantified within-lineage embedding separation between ecDNA-positive and ecDNA-negative tumors across cancers (Fig. 4e). Pronounced divergence was observed in UCEC, ACC, and LGG, providing classifier-independent evidence that ecDNA corresponds to a stable architectural shift in morphology space.

Finally, we asked whether Fuji could distinguish other mechanisms of focal amplification beyond ecDNA, including breakage-fusion-bridge (BFB) cycles, non-cyclic amplification, and linear amplification (Fig. 4f). Fuji achieved robust discrimination across all three mechanisms, with AUCs of 0.85 for BFB, 0.76 for non-cyclic amplification, and 0.73 for linear amplification, consistently outperforming baselines trained on the same embeddings. BFB-driven amplification, characterized by extreme chromosomal instability and architectural disruption, was detected with the highest accuracy, suggesting that distinct amplification mechanisms leave separable and mechanism-specific morphological imprints. Together, these results establish routine histology as a scalable readout of focal genomic amplification, enabling ecDNA-based risk stratification in settings where whole-genome sequencing is unavailable and supporting large-scale retrospective mining of archived tissue for ecDNA-driven disease biology.

### Inference of clinically actionable driver mutations and oncogenic pathway alterations

**Fig. 5:**
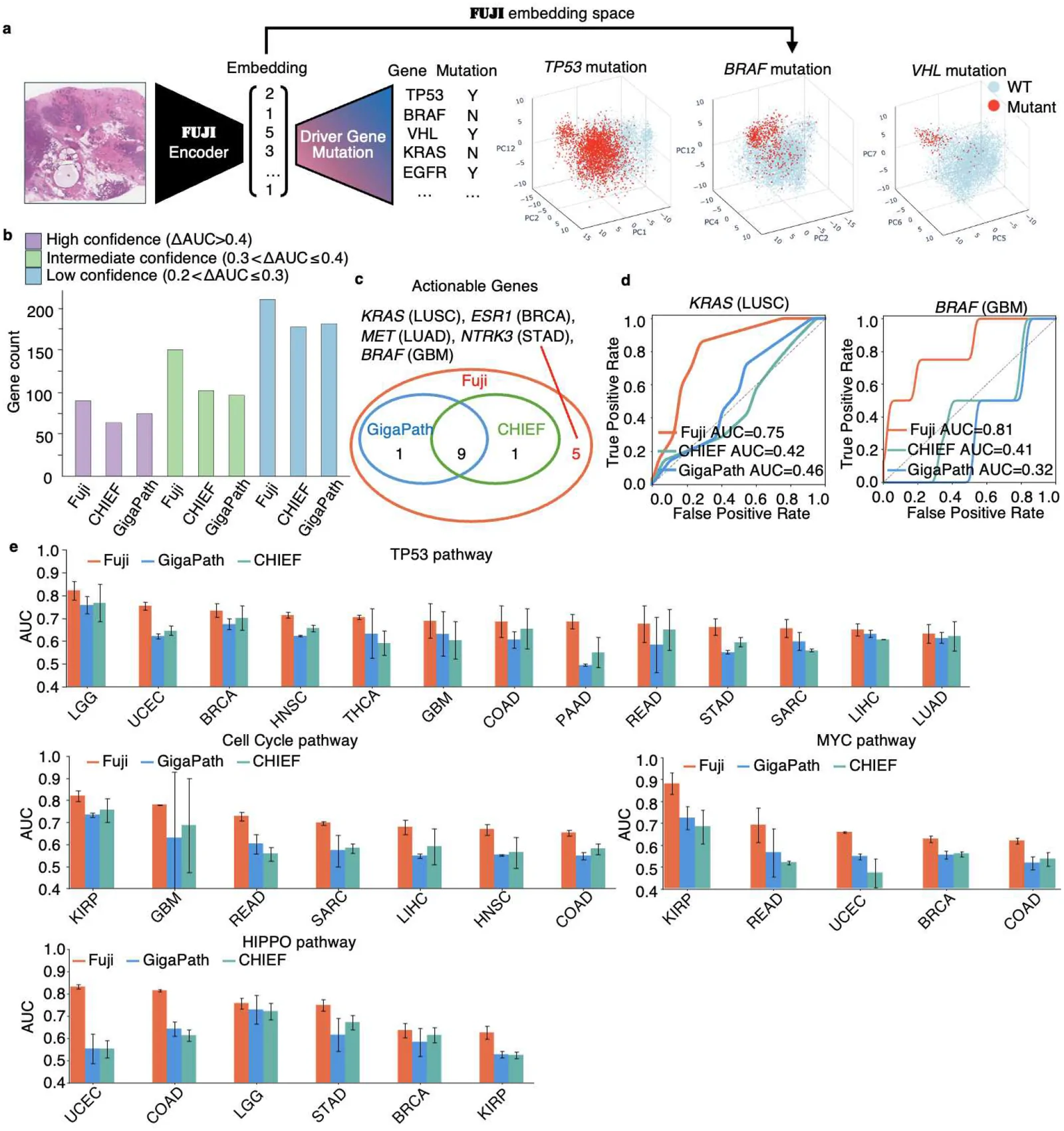
Driver mutation and oncogenic pathway inference from histopathology. **a**, (Left) Overview of the analysis. Whole-slide images are encoded into slide-level morphology embeddings, which are then used to infer driver-gene mutation status using linear probing on frozen embeddings. (Right) Visualization of embedding structure for representative canonical drivers. Three-dimensional PCA projections of slide embeddings are shown for TP53, BRAF, and VHL, with points colored by sequencing-derived mutation status (WT versus mutant), illustrating mutation-associated structure in morphology space. **b**, Systematic mutation predictability across tumor types. Number of driver gene-tumor type pairs exceeding a permutation-calibrated decoding-gain threshold (ΔAUC), stratified into low (0.2–0.3), intermediate (0.3–0.4), and high (>0.4) confidence tiers, comparing morphology embeddings to baseline pathology representations. **c**, Clinically actionable alterations. Overlap of predictable FDA-actionable driver gene-tumor type pairs across models; examples of actionable pairs uniquely recovered by the morphology-embedding approach are indicated. **d**, ROC curves for actionable driver prediction, including KRAS in LUSC and BRAF in GBM, with AUC values shown. **e**, Pathway-level alteration inference from morphology. AUC performance for predicting alterations in canonical oncogenic pathways (TP53, Cell Cycle, MYC, HIPPO) across indicated TCGA tumor types, comparing the morphology-embedding approach to baseline pathology models; bars show mean performance with error bars indicating variability across cross-validation folds.

We examined whether Fuji embeddings encode mutation-related morphological information at scale. Somatic driver mutations are primary determinants of cancer therapy selection, with multiple FDA-approved targeted agents matched to specific gene alterations. Principal component analysis of TCGA pan-cancer embeddings revealed that several components showed significant associations with sample-level somatic mutation status (Fig. 5a, Extended Data Fig. 6a). Canonical cancer gene mutations, including TP53, BRAF, and VHL exhibited structured clustering patterns across cancer types (Fig. 5a, Extended Data Fig. 6b), indicating that Fuji captures mutation-associated morphological signals with shared pan-cancer characteristics.

To systematically evaluate mutation predictability, we trained sample-level decoders to predict the mutation status of curated cancer driver genes using linear probing on frozen embeddings. Performance was quantified using a permutation-calibrated decoding gain (ΔAUC), defined as the difference between the observed cross-validated AUC and the mean AUC obtained from label-permuted baselines. Driver gene–tumor type pairs with ΔAUC>0.2 were considered predictable and stratified into low, intermediate, and high confidence tiers (Fig. 5b). Across all confidence tiers, Fuji identified substantially more predictable driver mutations than baseline models (Fig. 5b, Extended Data Fig. 7a), demonstrating more robust encoding of mutation-related information. Among all driver genes predicted by at least one model, 16 corresponded to genes with FDA-approved targeted therapies^43^, all of which were recovered by Fuji (Fig. 5c, Extended Data Fig. 7b). Notably, five clinically actionable driver gene–tumor type pairs were uniquely identified by Fuji (Fig. 5c), including KRAS in LUSC, ESR1 in BRCA, MET in LUAD, and NTRK3 in STAD, as well as BRAF in GBM, all corresponding to alterations with FDA-approved targeted therapies in one or more solid tumor contexts. These findings support the use of Fuji as a rapid morphology-based pre-screening tool for identifying candidates for targeted therapy, particularly in resource-limited settings where molecular sequencing is unavailable or delayed.

We further evaluated model performance at the level of oncogenic signaling pathways. Across four canonical pathways, TP53, Cell Cycle, MYC, and HIPPO, Fuji consistently outperformed baseline models in pathway-level alteration prediction across all evaluated TCGA tumor types with both FFPE and fresh-frozen specimens (Fig. 5e). These findings demonstrate that Fuji captures pathway-level oncogenic alterations that extend beyond individual gene mutations, supporting its utility for inferring functional molecular states directly from routine histology.

### Etiologic mutational processes leave reproducible architectural states

**Fig. 6:**
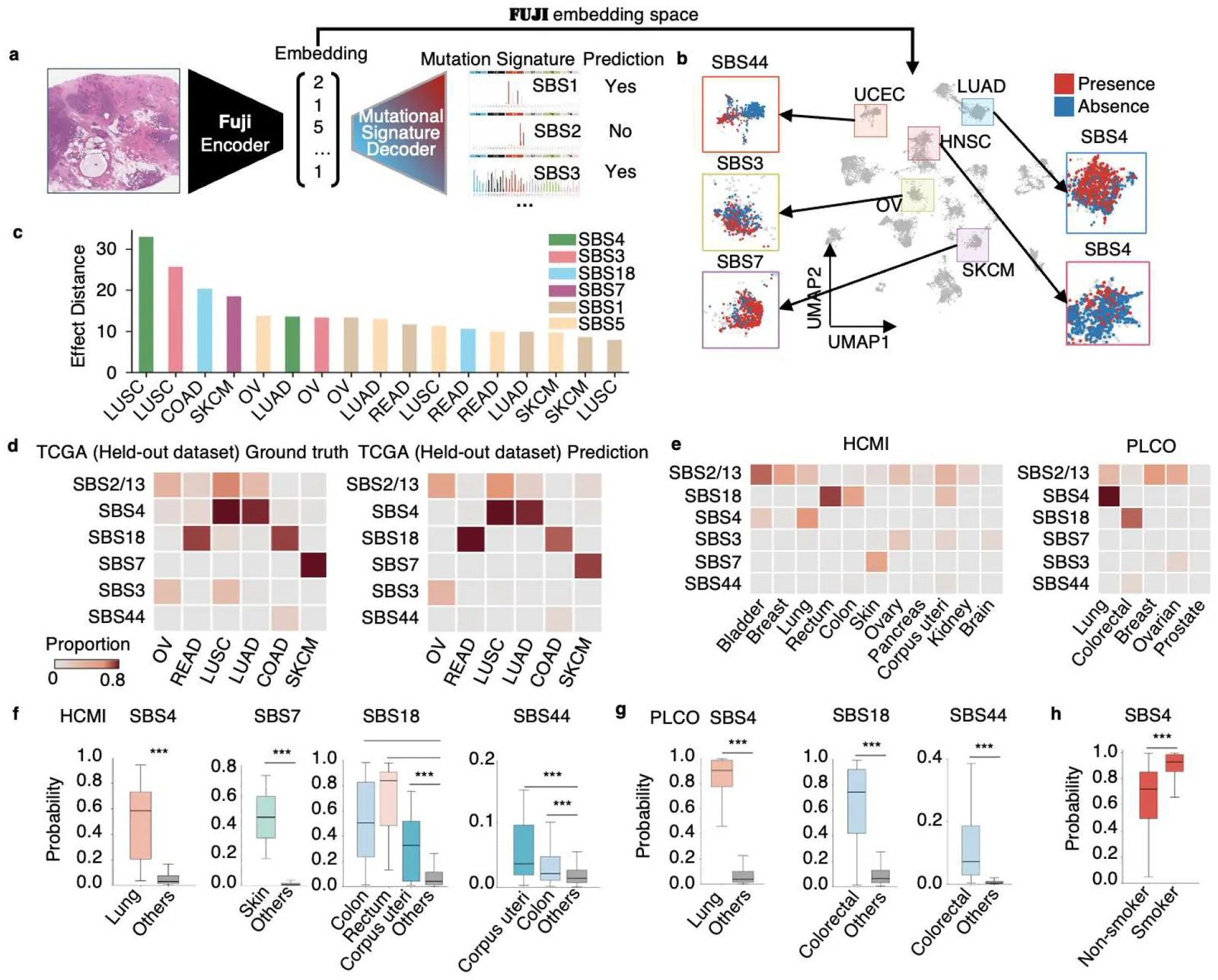
Mapping the global landscape of mutational signatures through histology. **a**, Decoding mutational signatures from histology. Workflow for mutational signature decoding, where slide embeddings are mapped to specific signature proportions. **b**, Discrete enrichment of mutational processes in morphological space. UMAP visualization of signature localization within the pan-cancer manifold. Insets highlight the coherent and separable regions occupied by signature presence (red) versus signature absence (blue) tumors for representative processes. **c**, Magnitude of mutational signature associated with morphology imprint. Fuji embedding effect distance of major mutational signatures. **d**, Concordance with genomic ground truth. Comparison of ground truth and Fuji predicted signature proportions in the TCGA held-out dataset. **e**, experimental and screening cohorts. Heatmaps displaying mutational signature decoder inferred signature proportions across various organs in the HCMI cohort and the PLCO cohort. **f** and **g**, Quantification of signature probabilities across tissue origins. Boxplots represent the predicted positive probabilities of representative signatures in HCMI and PLCO cohorts. **h**, Validation of etiological inference. Boxplot showing the predicted probability of the smoking-related signature SBS4 stratified by clinical smoking history in the PLCO cohort.

We next asked whether the latent manifold resolves the morphological consequences of diverse mutational processes, effectively encoding etiologic exposures as structured and reproducible architectural states. Single-base substitution (SBS) signatures capture distinct exogenous exposures and endogenous DNA repair processes (e.g., tobacco, ultraviolet light) and endogenous processes (e.g., DNA mismatch repair deficiency), providing a stringent test of whether mutagenesis leaves a systematic morphologic imprint. We trained a slide-level model to predict continuous signature probabilities from morphology embeddings (Fig. 6a). When projected onto the embedding manifold, several etiologic signatures exhibited discrete, non-random enrichment in morphological space (Fig. 6b): tobacco smoking-associated SBS4 segregated within lung tumors, ultraviolet-associated SBS7 localized within melanoma, and DNA mismatch repair–associated SBS44 was enriched within endometrial cancer. These patterns indicate that exposure-linked mutagenesis corresponds to recurrent tissue architectural states detectable at histologic resolution.

To quantify morphology signature coupling independent of classifier performance, we computed within-lineage embedding effect distances comparing high versus low signature activity (Fig. 6c). Effect sizes were signature- and lineage-dependent, with strong shifts for SBS4 in LUSC and SBS7 in SKCM, whereas lower-amplitude, clock-like processes (SBS1/5) showed weaker or more diffuse organization. Thus, selected etiologic processes project into measurable, cohort-reproducible architectural divergence, while other mutational processes may remain morphologically subtle.

We next evaluated prediction fidelity against sequencing-derived exposures in a held-out TCGA set (Fig. 6d). Across lineages, morphology-derived signature distributions recapitulated established etiologic structure, including enrichment of SBS4 in lung cancers, SBS7 in melanoma, and mismatch repair–associated signatures in gastrointestinal and endometrial tumors. Notably, these patterns emerged without cancer-type supervision during representation learning, arguing that the signal reflects architectural correlates of mutagenesis rather than re-encoding tissue identity. We then tested separability within lineage, holding tissue-of-origin constant. Within lung cancers, SBS4-high and SBS4-low tumors remained separable and concordant with sequencing-derived labels (Extended Data Fig. 8). Similar within-lineage structure was observed for SBS18 in colorectal cancer, SBS2/13 in bladder carcinoma, and SBS44 in COAD, supporting that signature-associated morphology captures process-linked variation beyond lineage clustering.

Finally, these associations generalized to independent cohorts. In HCMI^44^, morphology-derived predictions reproduced expected enrichments, including elevated SBS4 in lung tumors, SBS7 in skin-derived tumors, and mismatch repair–linked signatures in colorectal and endometrial tissues (Fig. 6e, f). In PLCO^45,46^, predicted signatures again showed lineage-consistent enrichment, with SBS4 elevated in lung cancers and mismatch repair–associated signatures enriched in colorectal tumors (Fig. 6e, g). Consistent with exposure biology, SBS4 probabilities were significantly higher in lung cancer from smokers than non-smokers (Fig. 6h), providing orthogonal validation that the architectural signal tracks etiologic history. Together, these results establish that key mutagenic exposures and repair defects are not morphologically silent, enabling routine histology to act as a scalable readout of mutational processes, and motivating morphology-based stratification of etiology-linked tumor biology when sequencing is unavailable or limited.

### Tissue segmentation for breast cancer pathology

**Fig. 7:**
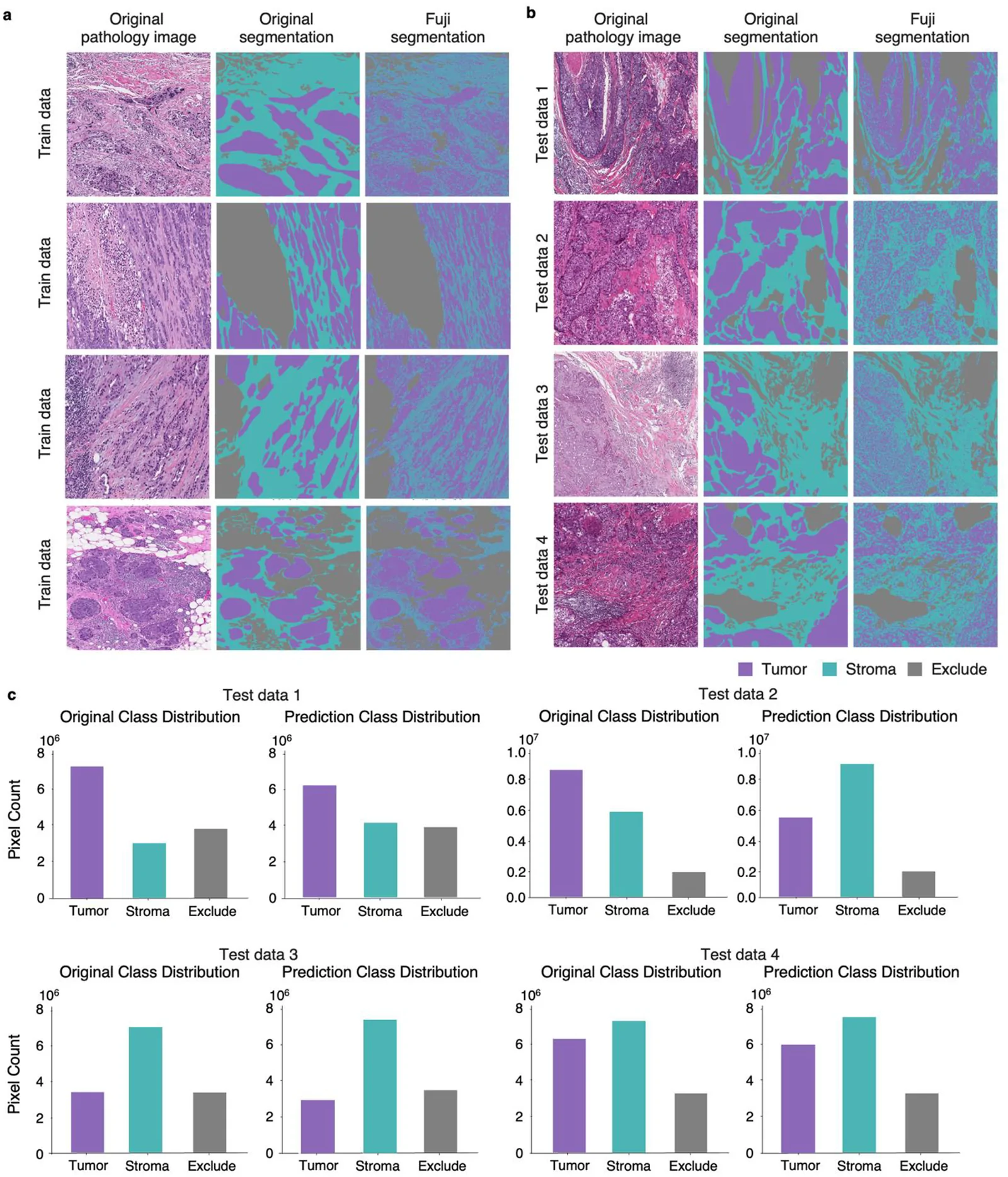
Evaluation of tissue segmentation. **a**, **b**, Representative tissue segmentation results for the training (a) and independent test (b) cohorts. Each row displays the original H&E-stained pathology image (left), the corresponding ground-truth mask (middle), and the model-derived segmentation (right). Color labels denote tumor (purple), stroma (teal), and excluded regions (grey). **c**, Pixel-level tissue class distributions. Bar charts show the total pixel counts for the three tissue categories across the four test cases presented in (b). Distributions are provided for both the original ground-truth annotations and the decoder-predicted masks.

To assess whether Fuji’s WSI encoder preserves spatially resolved tissue morphology, we paired the frozen Fuji encoder with a lightweight U-Net–based decoder to perform tissue segmentation. Without retraining the encoder, the decoder accurately reconstructed spatially coherent tumor, stroma, and exclusion regions that closely matched expert annotations across both training and independent test WSIs (Fig. 7a, b). Notably, Fuji-based segmentations recovered fine-grained architectural features, including elongated stromal fibers, infiltrative tumor fronts, and complex tumor–stroma interfaces, that were blurred or fragmented in the original annotations. Across all held-out test datasets, the predicted tissue class distributions closely matched ground-truth pixel distributions (Fig. 7c), indicating that Fuji embeddings preserve quantitative information about tissue composition in addition to qualitative morphological features. Together, these results demonstrate that Fuji’s WSI encoder encodes rich, spatially resolved tissue morphology, enabling accurate downstream dense prediction tasks and supporting its use as a general-purpose representation for spatial pathology analysis.

## Discussion

We present Fuji, a genome-informed multimodal pathology foundation model that transforms routine hematoxylin and eosin slides into a scalable readout of tumor genomic and evolutionary state. By integrating whole-slide images with transcriptomic profiles and mutational signatures during pretraining, Fuji couples tissue morphology to the molecular processes that shape it, enabling direct inference of genome-scale instability phenotypes, focal structural amplification, clinically actionable driver mutations, mutational processes, and immunotherapy response from histology alone. Across diverse cancer types, independent external cohorts, and both formalin-fixed paraffin-embedded and fresh-frozen specimens, Fuji consistently outperformed existing pathology foundation models. More fundamentally, by treating the learned embedding as a quantitative coordinate system of tissue morphology, we demonstrate that tumor genome evolution imposes reproducible, lineage-conditioned architectural constraints, establishing that histology encodes the structural and selective consequences of genomic evolution, not its full mutational spectrum.

The clinical implications of these findings are substantial. Comprehensive genomic profiling has become indispensable for modern oncology, guiding therapy selection, prognostic stratification, and clinical trial eligibility, yet access remains severely limited by cost, tissue requirements, turnaround time, and infrastructure. For most cancer patients worldwide, particularly in low- and middle-income settings and community practices, routine histopathology is the only molecular assay universally available. Fuji offers a path toward closing this gap. Our results suggest three distinct modes of clinical utility. First, Fuji may serve as a scalable pre-screening tool for clinically actionable alterations, prioritizing patients most likely to benefit from confirmatory sequencing and targeted therapy matching. Microsatellite instability inference with AUC values reaching 0.98 approaches the performance threshold relevant for companion diagnostic consideration, and the recovery of FDA-actionable driver gene–tumor type pairs across 16 distinct targets provides concrete therapeutic contexts. Second, Fuji enables morphology-based risk stratification for features currently inaccessible outside research settings, most notably extrachromosomal DNA, where our survival analyses in lower-grade glioma and endometrial carcinoma demonstrate that predicted ecDNA status carries independent prognostic information derived entirely from routine H&E. Third, Fuji unlocks the latent molecular value of archived pathology material. Hospitals and biobanks hold millions of FFPE slides from patients who were never sequenced; the ability to retrospectively infer genomic features from these slides could accelerate translational research, real-world evidence generation, and health-equity-focused epidemiology at a scale that prospective sequencing cannot match.

A key enabler of clinical translation is Fuji’s explicit unification of FFPE and fresh-frozen histology within a single genome-informed representation, which addresses a long-standing disconnect between clinical pathology practice and molecular genomics research. FFPE slides dominate routine clinical workflows and large retrospective cohorts, providing unmatched scale and outcome annotation but introducing fixation-related artifacts that can obscure subtle molecular signals. Fresh-frozen specimens, by contrast, better preserve nucleic acids and cellular ultrastructure and therefore underpin most high-quality transcriptomic and genomic profiling, yet remain scarce in clinical routine. Most existing pathology foundation models implicitly favor one modality, limiting either molecular fidelity or real-world applicability. By jointly modeling both preparation types and explicitly aligning their representations, Fuji suppresses protocol-specific variation while preserving genome- and disease-relevant morphology. This enables molecular supervision, derived primarily from fresh-frozen tissue, to inform interpretation of FFPE morphology at scale, a prerequisite for deploying genome-informed pathology models in clinical practice where fixation is the norm.

Beyond clinical utility, Fuji establishes conceptual advances for the biology of tumor evolution. Tumorigenesis is molecular in origin but architectural in outcome. Our results show that genomic processes are not randomly reflected in morphology; instead, they occupy structured regions of histologic representation space. Genome-scale instability features including MSI, CIN, HRD, WGD, and TMB consistently shift tumors within lineage-defined embedding neighborhoods. Focal amplifications, including ecDNA and breakage-fusion-bridge events, correspond to distinct architectural divergence. Selected mutational signatures, both endogenous and exogenous, localize to discrete morphological regions that replicate across independent cohorts including the population-based PLCO screening trial, where smoking-associated SBS4 predictions concord with recorded exposure history. MSI provides a particularly clear example of how an evolutionary regime can constrain tumors into a characteristic architectural configuration: MSI-positive tumors repeatedly concentrate within discrete subregions of colorectal, gastric, and endometrial morphology space, producing the largest within-lineage geometric shifts we observed. This pattern suggests that MSI functions as a tumor-wide evolutionary program, reshaping mutational burden, immune interactions, and stromal organization in a coordinated manner, rather than reflecting a few isolated genomic events. Genomic visibility, however, is selective. Clock-like signatures SBS1 and SBS5, which accumulate steadily in both normal and tumor tissues without a clear etiologic association, produce diffuse rather than coherent morphological distributions. This differential visibility is itself informative: it indicates that histology encodes the selective and structural consequences of genome evolution that reshape tissue organization, rather than recording the full mutational history. Defining these scopes and limits provides a principled framework for future development and interpretation of morphology-based genomic inference.

Methodologically, Fuji combines constrained multi-teacher distillation with multimodal multi-task molecular supervision to produce representations that are both robust and biologically structured. Freezing early visual layers preserves high-quality morphological features inherited from large-scale vision pretraining, while transcriptomic reconstruction and mutational signature prediction align morphology with functional cellular state and underlying mutational processes. Attention alignment constraints further ensure that genomic supervision is spatially grounded in histologically relevant tissue regions rather than distributed across slide-level features. The resulting unified embedding supports a modular ecosystem of task-specific decoders, enabling diverse molecular and clinical readouts to be inferred without retraining the foundation model. This architecture is intentionally extensible: new decoders can be added as new molecular annotations become available, without requiring repetition of the expensive pretraining stage.

Several limitations warrant consideration and motivate future work. First, genomic annotations used for supervision, including mutational signatures and genomic instability phenotypes, represent imperfect proxies for underlying biological processes and are subject to technical variability, algorithmic uncertainty, and context-dependent interpretation. Discordance at the individual-sample level may reflect ambiguity in ground-truth assignment rather than limitations of morphology-based inference. Second, the current framework operates at the whole-slide level and does not explicitly model intratumoral spatial heterogeneity of transcriptional or mutational processes, which may contribute to residual variability in prediction performance and which spatial genomics technologies are rapidly making tractable. Third, although attention-based constraints improve spatial grounding, the precise cellular and microenvironmental determinants mediating specific genome-linked morphological patterns remain incompletely understood; integration with single-cell and spatial transcriptomic profiling will be required to map these associations to specific cell populations and tissue niches. Fourth, while our evaluations span large, diverse, and geographically distinct cohorts including TCGA, CPTAC, GTEx, HCMI, and PLCO, prospective validation across multiple clinical institutions with varied staining protocols, scanner platforms, and patient populations will be essential before clinical deployment. The immunotherapy response findings in particular, derived from a limited cohort, should be regarded as hypothesis-generating and motivate dedicated prospective studies. Finally, regulatory pathways for morphology-based molecular inference remain underdefined; engagement with regulatory bodies and rigorous demonstration of performance equivalence with established assays will be necessary to translate these findings into approved clinical use.

Together, Fuji reframes routine histopathology as a quantitative and scalable readout of tumor genome evolution. By demonstrating that genomic and evolutionary states are systematically encoded in tissue architecture, and by delineating both the scope and the principled limits of this encoding, our results establish a foundation for democratizing molecular cancer profiling through the most widely performed assay in oncology.

## Methods

### Datasets for Fuji pretraining

Fuji utilized a multimodal corpus spanning histopathology, genomics, and transcriptomics across several large-scale cohorts. For foundational visual representation, we curated 60,546 H&E WSIs from the TCGA, CPTAC, and GTEx repositories, which were partitioned into non-overlapping 256 × 256 pixel patches at a resolution of 1.0μm per pixel (Supplementary Methods). During the initial contrastive pretraining phase, these preprocessed data were aligned with pre-extracted 768-dimensional CHIEF embeddings; matched FFPE and fresh-frozen slides from the same patient were also utilized to promote representational harmony across tissue preparations. In the subsequent multi-task pretraining phase, we focused on TCGA and CPTAC dataset and incorporated matched mutational signature profiles including SBS, DBS, and ID (Supplementary Methods), and paired transcriptomic embeddings derived from the OmiCLIP^17^ transcriptomic encoder. To facilitate independent evaluation, we implemented a 15% patient-level hold-out strategy on the TCGA cohort, reserving these cases for downstream clinical benchmarking and decoder validation.

### Model design and pretraining

For pretraining the multimodal Fuji foundation model, we incorporated two steps (Fig. 1a), including multi-teacher distillation for contrastive learning and multi-task learning combining masked image reconstruction, transcriptomic embedding reconstruction, and mutational signature inference for multimodal incorporation to learn genome-informed representations from WSI images, transcriptomics, and mutational signatures.

#### Contrastive pretraining

To develop unified histopathological representations, we implemented a multi-teacher distillation framework through contrastive learning. The Fuji encoder was initialized with pretrained GigaPath weights to inherit foundational morphological features. To balance knowledge retention with task-specific adaptation, we froze layers 1 to 11 of the transformer backbone while keeping the final (12th) layer trainable. Simultaneously, CHIEF was integrated as a second teacher model to diversify the morphological latent space. A lightweight MLP projection head (two linear layers, hidden dimension 768, dropout 0.1) was employed to bridge between the two teachers, mapping embeddings from the frozen CHIEF into the shared Fuji latent space. We implemented multi-teacher distillation via two parallel alignment tasks to form the contrastive loss ${ℒ}_{\text{con}}$ (Supplementary Methods). It aligned representations between Fuji and the CHIEF encoder via inter-teacher alignment ${ℒ}_{\text{inter}}$ while simultaneously aligning between FFPE and fresh-frozen for harmonious preparations of Fuji encoder through invar-teacher alignment ${ℒ}_{\text{invar}}$ (Supplementary Methods). Consequently, the objective function for contrastive learning is defined as a combination of slide embedding alignments:

(1) $${ℒ}_{\text{con}}={ℒ}_{\text{inter}}+{ℒ}_{\text{invar}},$$

#### Multi-task pretraining

In the second pretraining phase, the Fuji encoder transitioned from partial freezing to full pretraining, where all 12 transformer layers were unfrozen and jointly optimized. This phase utilized a multi-task learning framework designed to distill cross-modal information in histopathology, transcriptomics, and genomics. Training was performed exclusively on the TCGA cohort, maintaining the 15% patient-level hold-out strategy.

#### Masked image reconstruction task

To understand tissue architecture, we adopted a masked autoencoder strategy. For each WSI, 75% of the patch-level embeddings $e_{i}$ were randomly masked. The remaining tokens were encoded and passed to a ViT-base decoder (Supplementary Methods) to reconstruct the latent features of the masked set ℳ. The reconstruction objective was optimized using the MSE loss:

(2) $${ℒ}_{\text{img}}=\frac{1}{\left|ℳ\right|}\sum_{i\in ℳ}\frac{1}{d}\sum_{k=1}^{d}{\left({\overset{ˆ}{e}}_{i,k}-e_{i,k}\right)}^{2},$$

where d=1,536 is the preprocessed data embedding dimensionality (Supplementary Methods), and $e_{i,k}$ and ${\overset{ˆ}{e}}_{i,k}$ denote the ground-truth and reconstructed values of the k-th dimension for masked patch i, respectively.

#### Transcriptomic reconstruction task

To bridge morphological phenotypes with molecular states, we tasked the model with predicting transcriptomic embeddings derived from transcriptomic encoder. Patch-level embeddings $X=\left\{e_{1},\ldots ,e_{n}\right\}$ from the Fuji encoder were processed through TransNet, a projection network consisting of two fully connected blocks (hidden dimension 1,024, GELU activation, dropout 0.2). The global representation r was obtained via mean pooling: $\overset{ˆ}{r}=\frac{1}{n}\sum_{i=1}^{n}\text{TransNet}\left(e_{i}\right)$. The model optimized the $L_{1}$ loss between the predicted embedding $\overset{ˆ}{r}$ and the target transcriptomic embedding r derived from OmiCLIP:

(3) $${ℒ}_{\text{trans}}=\frac{1}{d}\sum_{k=1}^{d}\left|{\overset{ˆ}{r}}_{k}-r_{k}\right|,$$

where d=768 is the embedding dimensionality. This objective encourages the encoder to capture fine-grained morphological correlates that are predictive of transcriptomic profiles.

#### Mutational signature inference task

The inference task was optimized using a dual-loss objective comprising a signature classification loss and an attention alignment loss. To incorporate genomic hallmarks, we appended a 38-head attention-based transformer layer (Supplementary Methods) to the Fuji encoder. Each head was dedicated to one of K=38 independent mutational signatures $\left(s\in \left\{1,\ldots ,K\right\}\right)$. For each signature, the model generated a patch-level attention distribution to identify signature-specific morphological regions. To address class imbalance in the genomic annotations, we utilized cross-entropy loss for the classification of K mutational signatures:

(4) $${ℒ}_{\text{sig}}=\sum_{s=1}^{K}-\alpha_{y_{s}}\text{log}(p_{s,y_{s}}),$$

where $p_{s,y_{s}}$ is the predicted probability for signature s and the true class $y_{s}\in \{0,1\}$, and $\alpha_{y_{s}}$ represent the class-weighting factor of the ground-truth class.

To ensure that the localized morphological regions identified by the attention heads remained biologically consistent with the global informative regions, we implemented an attention alignment constraint (Supplementary Methods). We utilized the KL divergence to minimize the discrepancy between the global MIL attention distribution $a_{s}\in R^{n}$ of target n non-overlapping patches of each WSI and the learned signature-specific attention distribution ${\overset{ˆ}{a}}_{s}\in R^{n}$ for each head:

(5) $${ℒ}_{\text{attn}}=\sum_{s=1}^{K}\text{KL}\left(a_{s}\|{\overset{ˆ}{a}}_{s}\right),$$

The cumulative loss for the mutational signature inference task across all K heads is the mean of the individual signature losses and the attention alignment loss:

(6) $${ℒ}_{\text{ms}}={ℒ}_{\text{sig}}+{ℒ}_{\text{attn}},$$

Finally, the Fuji encoder learned a unified, genome-informed latent space by minimizing the total weighted loss for multi-task learning:

(7) $${ℒ}_{\text{multi}}=w_{1}{ℒ}_{\text{img}}+w_{2}{ℒ}_{\text{trans}}+w_{3}{ℒ}_{\text{ms}}.$$

where $w_{1},w_{2},w_{3}$ denote the respective weights for each task. This joint optimization strategy ensures that the resulting representations encapsulate complex correlations between histopathology, transcriptomics, and somatic genome evolution.

### Details of Pretraining Implementation and Settings

All experiments were conducted on a single NVIDIA H200 (141 GB HBM3e) GPU, where mixed-precision training (FP16) was leveraged to optimize computational throughput and memory efficiency. The contrastive learning was pretrained for 16 epochs with a local batch size of 20, and the multi-task learning phase was pretrained for 37 epochs with a batch size of 1 and gradient accumulation over 32 steps, yielding an effective batch size of 32. In the multi-task pretraining stage, training was conducted in two rounds using mutational signature profiles from different sources. The first round used WES-derived mutational signature profiles and was performed for 25 epochs with samples from the TCGA and CPTAC studies. The second round used WGS-derived mutational signature profiles and was conducted for 12 epochs within TCGA study. The Fuji encoder processes 1,536-dimensional input tile embeddings to generate 768-dimensional latent representations. It runs in Python v.3.9 with the following dependencies: torch v.2.8.0; torchvision v.0.23.0; ninja v.1.13.0; packaging v.25.1; numpy v.2.0.2; h5py v.3.14.0; pandas v.2.3.3; Pillow v.11.3.0; xformers v.0.0.32.post2; transformers v.4.57.6; timm v.1.0.24; einops v.0.8.1; fairscale v.0.4.13; scikit-learn v.1.5.2; scikit-image v.0.24.0; matplotlib v.3.9.4; seaborn v.0.13.2; tqdm v.4.67.1; pyparsing v.3.3.1; PyYAML v.6.0.3; huggingface-hub v.0.36.0; omegaconf v.2.3.0; fsspec v.2025.10.0.

### Trajectory inference

WSI images were obtained from a publicly available gastric cancer dataset comprising 104 WSIs from 44 patients, with expert pathologist–provided polygon annotations. The annotations capture the histological spectrum of gastric disease progression, including normal glands, chronic gastritis, lymphoid follicles indicative of Helicobacter pylori infection, precancerous lesions (chronic atrophic gastritis and intestinal metaplasia), and multiple gastric adenocarcinoma subtypes. Specifically, the annotated categories included nine pathological stages: normal gland, chronic gastritis, lymphoid follicles, chronic atrophic gastritis, complete intestinal metaplasia (IM), incomplete IM/low-grade dysplasia (LGD), well-differentiated tubular adenocarcinoma (tub1), moderately differentiated tubular adenocarcinoma (tub2), and papillary adenocarcinoma (pap).

For each WSI, all annotated polygons corresponding to each category were identified. To obtain representative regions, the largest annotated polygon for each category present in a slide was selected. The selected regions were cropped from the WSIs to generate region-level images for downstream analysis. In total, this procedure yielded 600 annotated regions spanning normal, inflammatory, precancerous, and malignant stages of gastric disease progression.

The 600 pathologist-annotated regions were encoded using the Fuji model to obtain high-dimensional feature embeddings. To visualize the structure of the embedding manifold, these embeddings were projected into a lower-dimensional space using Principal Component Analysis (PCA), and two principal components most strongly associated with histological progression were used for visualization.

To characterize progression along the manifold, a k-nearest-neighbor (kNN) graph was constructed using the top 50 principal components. A global ordering of regions was approximated by computing graph-based pseudotime $t_{i}$, defined as the geodesic distance from a root node selected at one extremity of the embedding manifold:

(8) $$t_{i}=\underset{p:r\to i}{\text{min}}\sum_{(u,v)\in p}d(u,v),$$

where d(u,v) denotes the Euclidean distance between neighboring regions in the embedding space.

A local progression vector field was then estimated by aggregating displacement vectors toward neighboring regions with increasing pseudotime:

(9) $$v_{i}=\frac{\sum_{j\in 𝒩(i)}w_{ij}\left(x_{j}-x_{i}\right)}{\sum_{j\in 𝒩(i)}w_{ij}},$$

where $x_{i}$ represents the embedding coordinates and $w_{ij}$ are distance-based weights.

The inferred vector field was then incorporated into the CellDancer diffusion framework to simulate velocity-driven trajectories across the manifold. Continuous pseudotime values were assigned by projecting regions onto the resulting trajectories. A smooth trajectory field u(x) was subsequently reconstructed by estimating the spatial gradient of the refined pseudotime:

(10) u(x)=∇t(x),

The reconstructed field was visualized using streamline plots to depict the inferred dynamics of gastric disease progression.

### Decoder Architecture and Framework

To bridge latent representations with clinical utility, the Fuji ecosystem integrates diverse downstream decoders (Fig. 1a, Extended Data Fig. 1b) analyzing mutational features, genomic instability, immunotherapy response, oncogenic pathway alterations, and tissue segmentation. These modules translate high-dimensional slide-level embeddings $s\in R^{768}$ obtained from Fuji encoder into interpretable phenotypic insights. The following sections detail the data curation, modeling strategies, and evaluation frameworks for each task.

### Mutational features

This module resolves multi-scale mutational landscapes directly from tissue morphology. We implemented a multi-head classification network trained on 22,686 TCGA WSIs to infer 4,132 hold-out data labels of mutational signatures (SBS, DBS, and ID) (Supplementary Methods), effectively mapping localized architectural hallmarks to specific mutagenic processes. Quantitative estimates of TMB were generated using RF trained on 22,686 TCGA WSIs, while task-specific binary classifiers were trained to identify discrete driver gene mutations (e.g., *TP53*, *KRAS*). Collectively, these decoders facilitate high-throughput digital genomic profiling, bridging the gap between histopathological phenotypes and nucleotide-level somatic alterations.

We first utilized ground-truth annotations derived from WGS and WES to define independent binary classification tasks, including SBS, DBS, and ID signatures. For each signature s, a specialized head (Supplementary Methods) projects the latent representation through a linear layer $l_{s}=W_{s}h+b_{s}$. To address class imbalance, we optimized the model using focal loss:

(11) $${ℒ}_{\text{sig}}^{\left(s\right)}=-\alpha_{y_{s}}{\left(1-p_{s,y_{s}}\right)}^{\gamma }\text{log}(p_{s,y_{s}}),$$

where γ=2.0 and $\alpha_{y_{s}}$ is the class-weighting factor. Biological fidelity was validated by recapitulating the mutational landscape across cancer types, with performance quantified via F1 and signature prevalence probability P(s∣t)=N(s=1,t)/N(t).

To establish a robust framework for predicting TMB, we curated a dataset of slide-level embeddings paired with binary clinical labels (high/low), applying stringent filters for cohort and class balance. Features were first standardized to zero mean and unit variance and reduced dimensions via SafePCA (Supplementary Methods), an adaptive dimensionality reduction framework determining the optimal number of principal components k. Standardized embeddings were then projected into a compact latent space $x\in R^{k}$ using a randomized singular value decomposition (SVD) solver. Here, we estimate the TMB morphological-genomic associations using an RF. Predictive performance was evaluated using 5-fold cross-validation to ensure patient-level independence, with the AUC and PR-AUC serving as the primary metrics.

For driver gene mutation, ground-truth labels were derived from non-synonymous somatic mutation calls of the TCGA dataset and binarized at the sample level (y=1 for mutated; y=0 for wild-type). Analysis was restricted to project–gene pairs with sufficient class support to ensure statistical reliability. We implemented a task-specific linear readout (Supplementary Methods) operating on slide-level embeddings s. The model maps the input directly to logits via a linear transformation:

(12) l=Ws+b,

where $W\in R^{2\times 768}$ and $b\in R^{2}$ represent weights and biases. To prevent overfitting and address class imbalances, the logistic regression models were optimized using $L_{2}$ regularization and class-balanced weights $\alpha_{c}=N/\left(n_{c}\cdot C\right)$ for c-th class in total class C based on N training samples and $n_{c}$ class samples. This linear probing strategy ensures that the performance reflects the inherent information content of the pre-trained representations regarding specific genomic drivers. Performance was measured via AUC, PR-AUC, and ΔAUC which is defined as the difference between observed AUC and a permutation-calibrated baseline (AUC=0.5). High-confidence signatures were identified using a threshold of ΔAUC>0.4.

### Genomic instability

Genomic instability annotations were obtained from curated TCGA Pan-Cancer Atlas datasets accessed through cBioPortal and TCGA-PanCanAtlas across multiple cancer types (Supplementary Table 1). Specifically, MSI annotations were obtained for COAD, STAD, and UCEC, comprising 1,408 patients. CIN annotations were obtained for COAD, ESCA, and STAD, including 1,061 patients. HRD status was derived for OV, BRCA, STAD, and UCEC, totaling 2,541 patients. WGD status is available for 5,413 patients across 12 cancer types.

For broad phenotypes, non-parametric random forest ensembles were utilized (Supplementary Methods). The RF classifiers were constructed as an ensemble of n=500 decision trees $\left\{T_{1},T_{2},\ldots ,T_{n}\right\}$. For an input embedding s, the class probability was estimated as the proportion of trees predicting the presence of the instability marker:

(13) $$P\left(y_{\text{broad}}=1|s\right)=\frac{1}{n}\sum_{i=1}^{n}I\left(T_{i}(s)=1\right),$$

where I(⋅) is the indicator function.

To resolve high-resolution structural genomic alterations including ecDNA, BFB, Non-cyclic complex rearrangements, and linear amplification, we implemented a suite of specialized decoders using a lightweight two-layer bottleneck architecture (Supplementary Methods) that maps slide-level embeddings s to a 48-dimensional latent representation $h=\text{ReLU}\left(W_{g}s+b_{g}\right)$ where $W_{g}$ and $b_{g}$ are the projection weights and bias of g-th decoder, ReLU(⋅) denotes the ReLU activation function, h is the latent hidden representation. These decoders identify distinct mechanisms of chromosomal crisis, such as ecDNA and BFB, via a classification head $l=W_{c}h+b_{c}$ optimized through a masked focal loss:

(14) $${ℒ}_{\text{focal}}=-\frac{1}{\left|M\right|}\sum_{i\in M}\alpha_{k}{\left(1-p_{i,k}\right)}^{\gamma }\text{log}(p_{i,k}),$$

where $W_{c}$ and $b_{c}$ are mechanism-specific classification weights and biases, M represents the set of samples with valid ground-truth labels, $p_{i,k}$ is the predicted probability for the true class k, and $\alpha_{k}$ and γ denote the class-weighting and focusing parameters, respectively. Decoders were assessed using lineage-specific prevalence, accuracy and AUC.

### Immunotherapy response

Clinical response data were curated at the patient level, where a responder was defined as exhibiting clinical response within 6 months of immunotherapy initiation. We implemented an optimized RF (Supplementary Methods) to predict clinical immunotherapy response of 32 patients (Supplementary Table 1) from slide-level embeddings s, estimating the probability of a specific outcome k∈{0,1} (where k=1 indicates therapeutic response) as the aggregate proportion of T decision trees predicting that class:

(15) $$P(y=k|s)=\frac{1}{T}\sum_{t=1}^{T}I\left(h_{t}(s)=k\right),$$

where y denotes the clinical response label, $h_{t}(\cdot )$ represents the prediction of the t-th individual decision tree, I(⋅) is the indicator function.

To address inherent class imbalances and ensure patient-level robustness, we utilized a cost-sensitive weighting factor $\alpha_{k}$ alongside a stratified group cross-validation strategy optimized by a heuristic balance score to minimize variance in class ratios and fold sizes (size of patients) across partitions:

(16) $$\text{Score}=\sigma^{2}\left({\text{ratio}}_{\text{pos}}\right)+\sigma^{2}\left({\text{ratio}}_{\text{neg}}\right)+0.5\sigma^{2}(\text{size}),$$

where ${\text{ratio}}_{\text{pos}/\text{neg}}$ are the proportions of positive and negative samples within each fold, and $\sigma^{2}(\cdot )$ represents the variance across cross-validation folds. Model stability was quantified through 25 distinct evaluations (5 random seeds × 5 folds). We report the average results of 5 seeds for AUC and PR-AUC.

### Oncogenic pathway alterations

Oncogenic pathway alteration labels were derived from a curated TCGA PanCancer Atlas dataset comprising 9,125 tumor samples across 33 cancer types, in which alterations in common canonical oncogenic signaling pathways (e.g., TP53, Cell-Cycle, and MYC pathways) were systematically annotated based on recurrent or known driver events (Supplementary Table 1). To map morphological hallmarks to functional molecular states, we developed a supervised decoding framework using a RF classifier optimized to predict oncogenic signaling pathway activation from high-dimensional slide-level embeddings (Supplementary Methods). The pipeline incorporates SafePCA to project standardized embeddings into a compact latent space based on the input data rank. Performance was evaluated within a pan-cancer framework to maximize class support and stratified by cancer type to assess tissue-specific heterogeneity. Metrics included AUC and PR-AUC.

### Tissue segmentation

We utilized 47 WSIs with high-resolution 4096 × 4096 pixel-level annotations of breast cancer for three biological categories: tumor, stroma, and exclude for tissue segmentation (Supplementary Table 1). To achieve high-resolution spatial quantification of the tumor microenvironment, we developed a multi-scale tissue segmentation framework utilizing a frozen Fuji encoder backbone coupled with a U-Net-style decoder (Supplementary Methods) to progressively reconstruct spatial resolution from latent representations for functional tissue classification. The model projects layer-specific latent embeddings $s_{l}$ into spatial feature maps $F_{l}=\text{Reshape}\left(W_{\text{proj}}s_{l}+b_{\text{proj}}\right)\in R^{768\times 16\times 16}$, which are optimized via a total objective ${ℒ}_{\text{total}}={ℒ}_{CE}+{ℒ}_{L2}+{ℒ}_{\text{entropy}}$ regularizing with $L_{2}$ and entropy penalty and featuring a dynamically weighted cross-entropy loss:

(17) $${ℒ}_{CE}=-\frac{1}{\left|𝒱\right|}\sum_{(i,j)\in 𝒱}w_{c(i,j)}\;\text{log}\frac{\text{exp}(l_{c(i,j)})}{\sum_{k=0}^{C-1}\text{exp}\left(l_{k}\right)},$$

where 𝒱 denotes the set of valid pixels, $l_{c(i,j)}$ is the logit value for the ground-truth class c at pixel (i,j), and $w_{c}$ is a dynamic batch-wise weight implemented to mitigate class imbalance between tumor and stroma compartments. This module provides the structural mask necessary to anchor all downstream genomic inferences in biologically salient tissue compartments.

Performance was quantified through pixel-level classification results on a reserved 10% (5 of 47 WSIs) test set across the valid tissue set 𝒱:

(18) $$\text{Accuracy}=\frac{1}{\left|𝒱\right|}\sum_{(i,j)\in 𝒱}1[\overset{ˆ}{y}(i,j)=\tilde{M}(i,j)].$$

where: 1[⋅] is the indicator function. $\overset{ˆ}{y}(i,j)$ is the predicted class label at pixel (i,j) using a threshold $\tau_{p}=0.5.\tilde{M}(i,j)$ is the ground-truth binary label.

## Supplementary Material

Supplementary Files

This is a list of supplementary files associated with this preprint. Click to download.

- SupplementaryMethods.docx
- ExtendedDataFigures.pdf

Extended Data Fig. 1 Fuji data and downstream tasks.

**a**, Fuji enables the deployment of specialized decoders for a comprehensive array of genomic and clinical tasks, including mutational features prediction (mutation signatures, TMB, and driver gene mutations), the detection of genomic instability hallmarks (MSI, HRD, ecDNA, CIN, and WGD), and the prediction of clinical outcomes such as immunotherapy response, oncogenic pathway alterations, and high-resolution tissue segmentation.

**b**, Utilization of the dataset across FFPE (blue) and fresh-frozen (red) WSIs. Data sources shown in the inset Sankey diagram include large-scale cohorts: TCGA, GTEx, CPTAC, with paired molecular data: 31,544 WES, 22,683 WGS, and 45,675 RNA-seq.

Extended Data Fig. 2 Anatomical lineage manifold.

**a**, Latent space of Fuji embeddings. UMAP projection of slide-level embeddings across the pan-cancer. Each point represents a single WSI, colored by cancer type. The clustering demonstrates the model’s ability to preserve biological variance and capture lineage-specific morphological signatures without explicit supervision.

Extended Data Fig. 3 Latent space of baseline foundation models.

**a** and **b,** UMAP of slide-level embeddings generated by GigaPath and CHIEF across the pan-cancer cohort. For each model, latent representations are visualized according to cancer type (left), anatomical tissue type (middle), and WSI preparation type (blue, FFPE; red, fresh frozen; right).

Extended Data Fig. 4 Prediction of mismatch repair gene mutations.

**a,** Cross-validated AUC values for predicting mutations in mismatch repair genes (*MLH1*, *MSH2*, *MSH6*, and *PMS2*) across TCGA projects, including COAD, STAD, and UCEC, using FFPE samples. Bars represent the mean AUC across five cross-validation folds, and error bars indicate the variability across folds.

**b,** Cross-validated AUC values for predicting mutations in mismatch repair genes (*MLH1*, *MSH2*, *MSH6*, and *PMS2*) across TCGA projects, including COAD, STAD, and UCEC, using fresh frozen samples. Bars represent the mean AUC across five cross-validation folds, and error bars indicate the variability across folds.

Extended Data Fig. 5 Prediction of global mutational burdens.

**a** and **b,** Bar charts comparing the AUC of Fuji and baseline models for the prediction of silent tumor mutational burden (silent TMB, top, a) and non-silent tumor mutational burden (non-silent TMB, bottom, b) across various malignancy lineages. Error bars indicate standard deviation between FFPE and fresh-frozen.

Extended Data Fig. 6 Pan-cancer associations between somatic mutations and embedding structure.

**a,** The correlation between selected somatic mutations and PCs derived from Fuji embeddings.

**b,** TCGA projects projected onto the three principal components. Each point represents a sample, colored by cancer type (TCGA project).

Extended Data Fig. 7 Predictable actionable driver mutations.

**a,** Pairwise differences in the number of uniquely predictable driver gene–tumor type pairs between models. (Left) high-confidence pairs (ΔAUC>0.4); **middle**, intermediate-confidence pairs (0.3<ΔAUC≤0.4); (Right) low-confidence pairs (0.2<ΔAUC≤0.3). For the intermediate- and low-confidence tiers, pairs present in higher-confidence tiers were excluded to ensure non-overlapping comparisons. Matrix entries represent the number of pairs uniquely identified by the row model but not by the column model.

**b,** FDA-approved actionable driver genes that are predictable by Fuji, CHIEF, and GigaPath, stratified by confidence tiers: high confidence (purple), intermediate confidence (green), and low confidence (blue).

Extended Data Fig. 8 Systematic validation of mutational signatures.

**a,** Slide-level prediction accuracy and overlap. Venn diagrams illustrate the quantitative overlap between TCGA hold-out ground-truth labels (blue) and predictions (red) from the mutational signature decoder for selected signatures.

## Data Availability

The Whole Slide Images (WSIs) and associated multi-omics data for pretraining and validation were obtained from the TCGA, GTEx, and CPTAC repositories. Data for trajectory analysis were sourced from the gastric cancer dataset. And datasets used in decoders evaluating the findings of this study include external cohort HCMI and PLCO, region-level pathology images, gene annotations, immune response results, survival analysis, and tissue segmentation. All relevant datasets are provided in Supplementary Table 1.

## Code availability

The full source code for the Fuji encoder was implemented in Python and is publicly available at the supplementary source code file Fuji-Fuji_FM.zip. To facilitate the use of our framework, we have provided comprehensive tutorials for WSI preprocessing and slide-level inference at this link. Pre-trained model weights can be accessed via https://drive.google.com/file/d/1Z4puEA3jGxe-SpCmafQXy8_OM4YaeDiT/view. All evaluation results and reproduction scripts are hosted within the main repository. Finalized source code and pre-trained weights will be officially released on GitHub and Hugging Face upon the acceptance of this manuscript.
